# TMT-based proteomics analysis of the cerebral cortex of TauT knockout rats

**DOI:** 10.1186/s12953-022-00189-z

**Published:** 2022-04-25

**Authors:** Yiming Xia, Xiaoling Huang, Lidong Mo, Chen Wang, Weijia Fan, Huiling Huang

**Affiliations:** 1 Clinical College of Neurology, Neurosurgery and Neurorehabilitation, Tianjin Medical University, Tianjin, 300070 China; 2 Tianjin Key Laboratory of Cerebral Vascular and Neurodegenerative Diseases, Tianjin Neurosurgical Institute, Tianjin Huanhu Hospital, Tianjin, 300350 China

**Keywords:** Taurine transporters, Taurine, Proteomics, Knock out

## Abstract

**Background:**

Taurine serves a variety of nutritional and physiological roles, and it is mostly transported in cells via taurine transporter (TauT). The effect of taurine transporter in cerebral cortex is still unknown. We employed TMT label-based proteomics to find differences in proteins in the cerebral cortex of TauT knockout rats in this investigation. The goal of this research was to see how TauT deletion affected protein alterations in brain tissue and to see if there was a new research area for TauT.

**Methods:**

The cerebral cortex of TauT knockout rats and wild-type control rats were analyzed using TMT-based proteomics, and differentially expressed proteins were analyzed by bioinformatics analysis means such as GO and KEGG, the association between the proteins was found by PPI, and biologically significant and interesting proteins were selected for verification by WB and immunohistochemistry.

**Results:**

There were total of 8275 proteins found, but only 35 differentially expressed proteins were identified (27 up-regulated and 8 down-regulated), and gene ontology (GO) and Kyoto Encyclopedia of Genes and Genomes (KEGG) analyses were performed to predict the biological pathways and functional classification of the proteins. The results show that these differentially expressed proteins are mainly enriched in lysine degradation, cell cycle, chronic myeloid leukemia, and longevity regulating pathways-multiple species, renal cell carcinoma, pathways in cancer, etc. To verify the proteomic data, we analyzed the expression of Annexin6 and Pik3r2 by western blotting and immunofluorescence. The results are consistent with proteomics, which proves the reliability of our proteomics data.

**Conclusion:**

Through TMT-based proteomics, we have a comprehensive understanding of the effect of TauT knockout on the changes of other proteins in the cerebral cortex, providing new evidence for further understanding the function of TauT.

## Introduction

Taurine is a sulfur-containing-amino acid. It does not participate in the formation of proteins, but it has a wide range of physiological functions in the body [1]. Taurine levels in brain tissue are very high, up to 40 mM [2]. It is mainly involved in the regulation of cell volume in the brain and neuromodulation by depolarizing the release of taurine [3]. In addition, taurine can promote the development of the brain and play a protective role in inflammation, apoptosis, and oxidative stress during brain injury [4–6]. Taurine transport is mainly achieved by taurine transporters (TauT). In normal tissues, the intracellular concentration of taurine is much higher than the extracellular concentration. The active transport of taurine through the TauT requires 23 sodium ions and one chloride ion for transport into the cytoplasm [7]. The construction of the TauTKO model has been reported in other literature. Ito et al. constructed TauTKO rats by knocking out exon 2–4 of the TauT gene, while Heller-Stilb et al. constructed TauTKO rats by knocking out exon 1 of the TauT gene [8, 9]. According to the report, TauT knockout can damage the ammonia detoxification of the mouse liver and also lead to weight loss and heart and skeletal muscle abnormalities [8]. In addition, ammonia transport of RhBG in the liver of 3-month-old TauTKO mice was impaired, and glutamine synthetase in 12-month-old TauTKO mice was inactivated due to tyrosine nitriation [10]. There are few reports on the effects of TauT on brain tissue. Qvartskhava N et al. just found that TauT knockout triggered RNA oxidation and protein tyrosine nitriation in the brain [10]. In this study, we successfully obtained TauT systemic knockout rats by CRISPR/Cas9 knockout of exon 5 of TauT [11], and we will focus on the effects of TauT knockout on brain tissue.

For decades, the function of taurine has been widely understood, and the exploration of taurine seems to have reached a bottleneck. In recent years, with the development of mass spectrometry, proteomics has become a new method to explain the function of proteins [12]. Proteomics is important for early diagnosis, prognosis, and monitoring of disease development. In addition, it plays an important role in drug development as a target molecule.Proteomics is the characterization of proteomics, including the expression, structure, function, interaction, and modification of proteins at any stage [13]. In this article, we used proteomics to examine the differentially expressed proteins in the cerebral cortex of TauTKO and wild-type rats, chose proteins of interest using GO and KEGG analyses, and double-checked the results with further experiments. Among 35 differentially expressed proteins, Annexin6 and Pik3r2 were chosen for expression verification. Pik3r2 was also identified to be an important proto-oncogene involved in tumor development. Many studies have indicated that reducing TauT causes tumor growth to be postponed, albeit the mechanism remains unknown. As a result, our findings may provide potential treatment targets as well as new insights into the mechanism of TauT tumor inhibition.

## Materials and methods

### Animals

Adult healthy TauTKO rats (constructed by our laboratory) and wild-type SD rats (WT rats, purchased from Huafukang Biotechnology company, Beijing, China) weighed between 250 and 300 g (male and female unlimited). The experiment was divided into two groups: the TauTKO group and the WT group. Each group of rats was randomly selected.

All rats were raised in an SPF environment where the temperature was about 25 °C, the air humidity was kept at 50%, and the light and dark cycle were 12 h/d. All the experimental animals were examined by the experimental Animal Ethics Committee of the Institute of Radiology, Chinese Academy of Medical Sciences.

### Sample collection

Rats were anesthetized for euthanasia and sacrificed with 10% chloral hydrate (0.3 mL/100 g). The cerebral cortex was removed rapidly and snap-frozen into liquid nitrogen before storage at -80 °C until needed.

### TMT labeled quantitative proteomics

#### Sample preparation

Three cortical samples from each group were taken for proteomic analysis. Then, 400µL of 8 M urea was added to the sample, and the protease inhibitor was added at 10% of the lysate. After centrifuging at 14000 g for 20 min, the supernatant was collected. The protein concentration was determined by the Bradford method.

Then 100 µg of protein was extracted from each sample for reduction. A 100mM solution of dithiotreitol (DTT) was added and incubated at 30 °C for 1 h, then alkylated with sufficient iodoacetamide (IAM) for 1 h at room temperature. Add 25 mM ammonium bicarbonate buffer and dilute 4 times. Trypsin (trypsin: protein = 1:50) was added and incubated overnight at 37℃. Finally, high urea was removed by C18 filter cartridge, and the samples were dried by vacuum centrifugation.

#### TMT labeling

The TMT reagent was incubated to room temperature. Add 41 μL (0.8 mg/ tube) anhydrous ethanol to TMT reagent and mix. Add 41 μL TMT reagent to 100 μg digested sample. Then, the reaction was terminated by shaking, centrifugation and incubation at room temperature for 1 h and in 5% quenching agent (8 μL) for 15 min. The samples were freeze-dried and preserved.

#### Peptide separation by high pH reversed-phase HPLC

Gradient elution was performed on mobile phases A (2% acetonitrile, pH adjusted to 10.0 with ammonium hydroxide) and B (98% acetonitrile, pH adjusted to 10.0 with ammonium hydroxide). The lyophilized powder was dissolved in solution A and centrifuged at 14000 g at room temperature for 20 min. Firstly, RIGOL L-3000 double gradient high performance liquid chromatography (HPLC, Rigol Technologie, Co. LTD, China) was used with the Agela Durshell-C18 column (4.6 × 250 mm i.d., 5 μm, 100 Å). The polypeptide was isolated. The column temperature is set at 45 °C. Solvent gradient: 5% B, 0 min; B5-8%; B. 30 min; B. 27 min; 32–95%-B, 6 min; 95–5%, 4 min. The eluent was detected under UV at 214 nm. Every minute, one tube was collected and fused into 10 fractions. All fractions were dried under a vacuum.

#### LC–MS/MS analysis

The dry polypeptide was dissolved in 10μL A solution and centrifuged at 14000 RPM for 20 min. The 1 µg of supernatant was injected into a nano-UPLC-MS/MS system consisting of a Nanoflow HPLC system (Easy-NLC 1000 system, ThermoFisher Scientific) and an Orbitrap Fusion Lumos Mass Spectrometer (ThermoFisher Scientific). Each sample was injected into the column and eluted in gradients from 6 to 38 % solution B in 70 min, from 38 to 100 % B in 1 min and 100 % B in 10 min at 200 nL/min (Solvent A, 100 % H2O; Solvent B, 80 % acetonitrile; both containing 0.1% (v/v) formic acid). The ion source of the mass spectrometer operates nanospray flex (NSI), the spraying voltage is 2.3 kV and the ion transport capillary temperature is 320 °C.  A precursor MS1 scan (m/z 300–1400) was acquired in the Orbitrap at the nominal resolution setting of 120,000 (200 m / z )with an automatic gain control (AGC) target of 5E5 and a maximum ion injection time of 100 ms. The highest rich top 40 data-dependent mode was analyzed by higher energy selection and dispersed collision dissociation (HCD) and MS/MS. MS/MS spectra were collected in the Orbitrap (50,000 resolution) with an AGC target of 2.5E4 and a maximum ion injection time of 86 ms. The peptide fragmentation collision energy was set to 32%. The dynamic discharge resistance range is set to 18s.

#### The data analysis

LC–MS/MS raw data were searched and quantitative analyzed from protein sequence database (Uniprot_RAT_2020_08). The mass spectrometry analysis of TMT was completed by Orbitrap Fusion mass spectrometry, and the original mass spectrometry files generated were processed by Proteome Discoverer 2.4. The searched parameters are set as follows: enzyme:trypsin; static modification: carbamidomethyl (C); dynamic modification: Oxidation(M), acetyl (protein N-terminal); species: rat; precursor ion mass tolerance: ± 15 ppm; Fragment ion mass tolerance: ± 0.5 Da; max missed cleavages: 2. the differentially expressed proteins (DEPs) were satisfied the following conditions: average ratio-fold change > 1.2, as well as p-value < 0.05.

#### Bioinformatics analysis

A global heat map was exerted to exhibit DEPs. The Gene Ontology (GO, which includes biological process (BP), cellular component (CC), and molecular function (MF)) annotation of the identified proteins was derived from the UniProt database. The KEGG database (https://www.genome.jp/kegg/) was used to perform the enrichment analysis of pathways. A study called STRING (https://www.string-db.org) was conducted to create protein–protein interaction (PPI) networks, which were used to identify interactions.

### Western blot

The cerebral cortices of TauTKO rats and WT rats were collected and total proteins were extracted using efficient radioimmunoprecipitation assay (RIPA) lysate kit (solarbio, R0010, China). The protein concentration was determined using the BCA kit (solarbio, PC0020, China). 40 μg proteins were loaded and separated on 10% polyacrylamide SDS-PAGE gel and then were transferred to polyvinylidene fluoride (PVDF) membranes. The membranes were blocked with 5% nonfat dry milk in TBST solution for 2 h at room temperature. Then, membranes were incubated with the rabbit anti-TauT(1:1000, Santcruz biotechnology, USA), mouse anti-GAPDH (1:10000, Proteintech, 60004–1-lg, USA) overnight at 4 °C. After being washed for 3 times with TBST (10 min each time), membranes were incubated with horseradish peroxidase (HRP) conjugated goat anti-mouse IgG secondary antibody (1:10000, ZB-2305, bs-10966R, China) and goat anti-rabbit IgG secondary antibody (1:10000, ZSGB-BIO, ZB-2301, China) for 90 min at room temperature. Then they were washed for 3 times with TBST (10 min each time). Membranes were incubated with Enhanced chemiluminescence (ECL)reagent (Merck Millipore, Germany). protein expression was analyzed with a gel imaging system (Fusion FX7, ViIbert Lourmat, France). The relative expression of the target protein was the ratio of the gray value of the target protein band to that of the internal reference protein band.

### Immunofluorescence

After PBS perfusion, the brain tissue of rats were fixed with 4% paraformaldehyde for 24 h, dehydrated with 30% sucrose to the bottom, embedded with OCT glue, quickly frozen with liquid nitrogen and preserved at -80 ℃.The frozen slicer was used to cut into 6 micron slices, fixed with 4% paraformaldehyde for 15 min, washed with PBS for 3 times, 3 min each, and 0.5% Triton for 30 min, washed with PBS for 3 times, 3 min each, and sealed with goat serum for 30 min. rabbit anti-Annexin6 (1:100, Proteintech, 12542–1-AP, USA) and mouse anti-Pik3r2 (1:100, Proteintech, 67644–1-Ig, USA)) primary antibodies were incubated overnight, washed with PBS 3 times for 3 min each, and goat anti-rabbit(1:50, Proteintech, SA00003-1, USA)) and goat anti-mouse(1:50, Proteintech, SA00003-2, USA)) secondary antibodies were incubated at 37° for 1 h, washed with PBS 3 times for 3 min each, stained with DAPI and sealed.The results were observed under a confocal microscope (LSM 800, Zeiss, Germany), and 5 fields at high magnification (200 ×) were randomly selected for observation.

### Statistics

All data were expressed as mean ± standard deviation (SD). Statistical analysis was conducted by SPSS software (version 17.0). Results were compared with an independent t-test. A p-value of 0.05 was considered statistically significant.

## Results

### Proteomic analysis to identify and quantify proteins

**Fig. 1:**
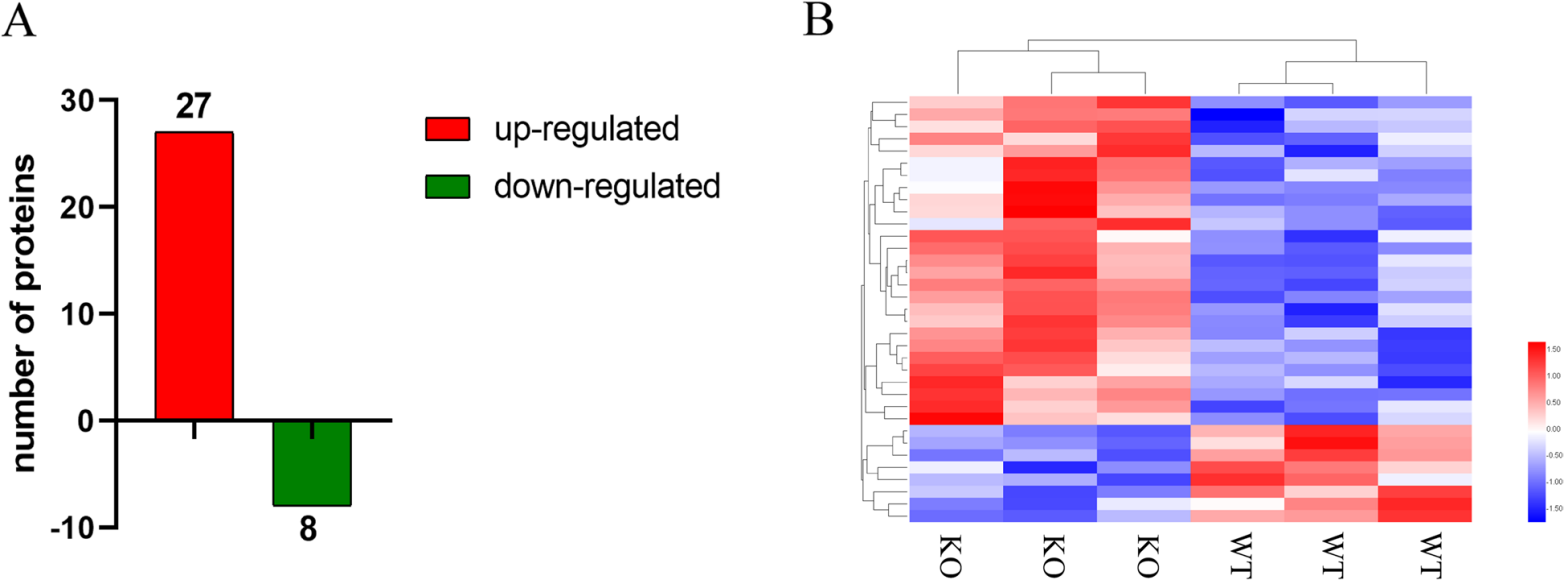
Total differentially expressed proteins. **A** Total number of identified DEPs from TauTKO/WT groups; **B** Hierarchical cluster analysis of DEPs

In this experiment, in order to investigate the effects of TauT deficiency on the cerebral cortex of rats, TMT-Base proteomics was used to study the changes in all protein expressions. Here, a total of 8,275 proteins were identified (FDR < 0.01), while only 35 differential proteins were detected in TauTKO rats and WT rats, among which 27 protein expressions were upregulated and 8 protein expressions were downregulated (Fig. 1; Table 1 and 2).

**Table 1:** upregulated protein

Gene name	Protein description	score	Sequence coverage	Unique peptides
Rbx1	Ring-box 1	34.17	13	3
Ogfrl1	Opioid growth factor receptor-like protein 1	25.14	11	4
Bin2	Bridging integrator 2	81.1	16	5
H1.5	Histone H1.5	33.86	10	2
Purb	Transcriptional activator protein Pur-beta	338.13	64	1
Lrrc8d	Volume-regulated anion channel subunit LRRC8D	25.28	9	6
Hdac2	Histone deacetylase 2	41.47	15	3
Igsf1	Immunoglobulin superfamily member 1	36.25	7	7
Krt27	Keratin, type I cytoskeletal 27	45.73	8	2
Txndc15	Thioredoxin domain-containing protein 15	24.52	12	3
Bbs7	Bardet-Biedl syndrome 7 protein homolog	12.84	8	4
Iqcb1	IQ calmodulin-binding motif containing 1 (Predicted), isoform CRA_a	21.18	5	3
Rnasel	Ribonuclease L	27.58	11	8
Bag3	BAG cochaperone 3	69.42	20	8
Pcdhga6	Protocadherin gamma subfamily A, 6	20.25	5	3
Hint3	Histidine triad nucleotide-binding protein 3	134.62	46	7
Sqle	Squalene monooxygenase	14.82	9	4
Peg3	Paternally-expressed 3	19.09	3	3
Anxa6	Annexin	986.5	71	51
Aass	Alpha-aminoadipic semialdehyde synthase, mitochondrial	46.1	14	13
Snx13	Sorting nexin 13	16.48	6	4
Cd200	OX-2 membrane glycoprotein	178.48	18	1
Paf1	RNA polymerase II associatedfactor 1 homolog	36.55	22	9
Bcat2	Branched-chain-amino-acid aminotransferase, mitochondrial	29.53	17	5
	2-phospho-D-glycerate hydro-lyase	28.32	8	1
Lztr1	Leucine-zipper-like transcription regulator 1	16.22	3	2
Ctdsp1	Protein-serine/threonine phosphatase	9.39	8	2
Dync2h1	Cytoplasmic dynein 2 heavy chain 1	3.71	0	2

**Table 2:** Downregulated protein

Gene name	Protein description	score	Sequence coverage	Unique peptides
Focad	Focadhesin	40.08	4	6
Mag	Myelin-associated glycoprotein	380.97	26	1
Slc6a11	Sodium- and chloride-dependent GABA transporter 3	293.69	13	8
Pik3r2	Phosphatidylinositol 3-kinase regulatory subunit beta	55.07	11	6
Map1a	Microtubule-associated protein 1A	4815.46	58	2
Tmlhe	Trimethyllysine dioxygenase, mitochondrial	29.97	18	7
Dbndd2	Dysbindin domain-containing 2	17.82	20	2
Ccdc178	Coiled-coil domain-containing 178	6.85	1	1

### Gene ontology (GO) analysis of differentially expressed proteins

**Fig. 2:**
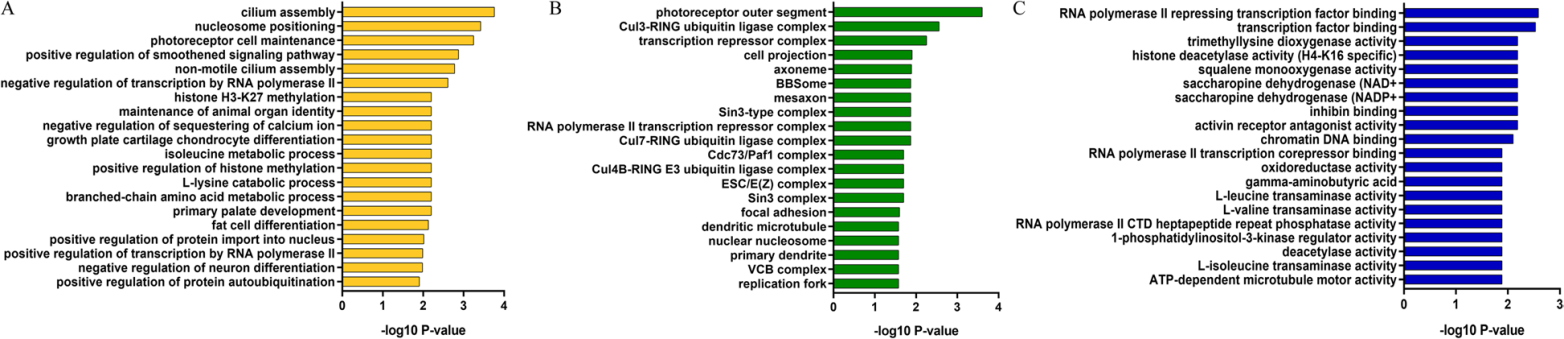
GO analysis of differentially expressed proteins. **A** Biological Processes; **B** Cellular Components; **C** Molecular Functions

From GO analysis, we understand the activity, localization, and function of differentially expressed proteins through biological processes, cellular components, and molecular functions. We selected the top 20 for analysis (Fig. 2). In terms of biological processes, the biological processes include Cilium Assembly, nucleosome positioning, photoreceptor cell maintenance and positive regulation of smoothened signaling pathway, etc. On cellular Component, it mainly involves photoreceptor outer segment, Cul3-ring ubiquitin Ligase complex, transcription repressor Complex, cell projection, etc. Regarding molecular functions, the main functions are RNA polymerase II Repressing transcription factor binding, transcription factor binding, Trimethyllysine Dioxygenase activity and Histone Deacetylase activity (H4-K16 specific), etc.

### Analysis of protein–protein interaction (PPI) network between differentially expressed proteins

**Fig. 3:**
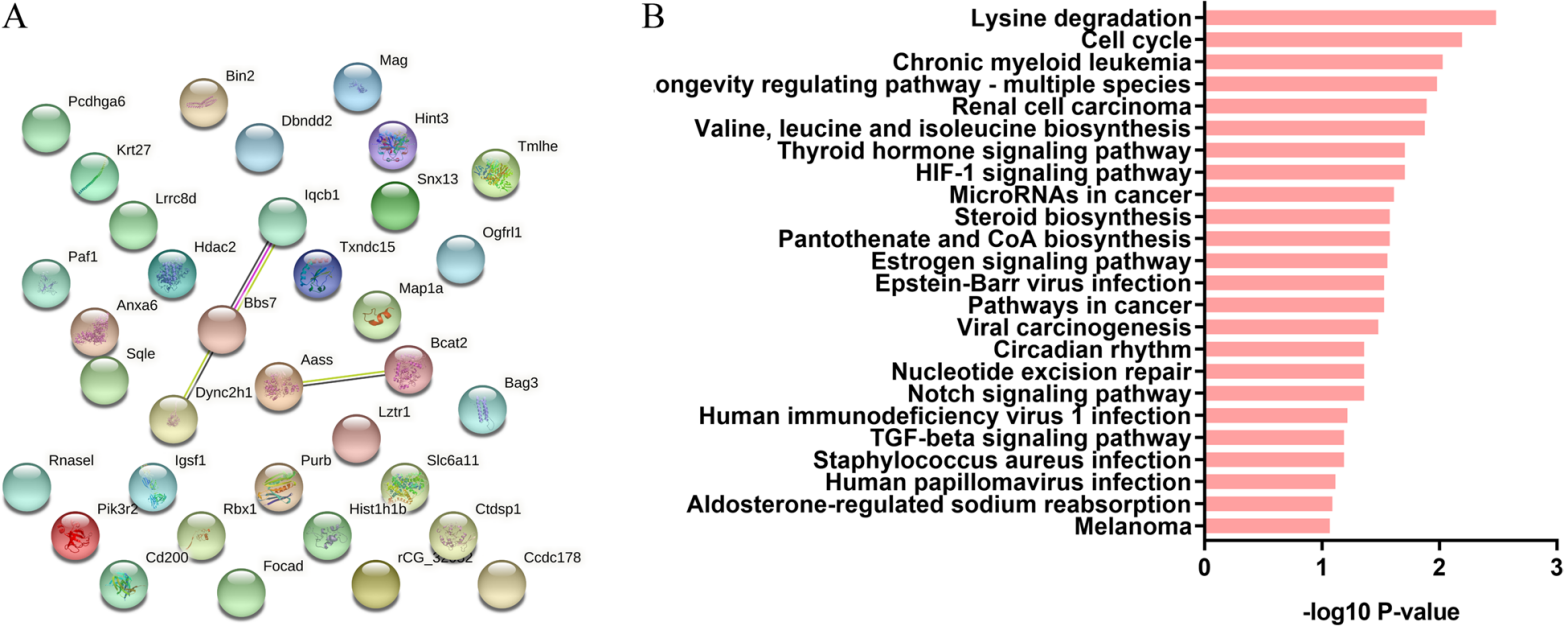
PPI analysis and KEGG pathway analysis. **A** PPI analysis of differentially expressed proteins; **B** KEGG pathway analysis of differentially expressed proteins

From GO analysis, we generally understand the classification of differentially expressed proteins. In order to further understand the interaction between differentially expressed proteins, PPI analysis was performed on 35 differentially expressed proteins (Fig. 3A). We found that most of the 35 proteins did not interact with each other; only Dync2h1, Txndc15, and Lqcb1 might interact. Dync2h1, Txndc15, and Lqcb1 all participate in cilium assembly, but the interaction between Aass and Besat2 is poorly understood. These results suggest that the up-regulation and down-regulation of differential proteins caused by TauT knockout may be the result of intracellular taurine deficiency rather than the direct effect of TauT. This is consistent with the broad physiological effects of taurine. For example, we know that Lrrc8d is necessary for volume regulated anion channel (VRAC) formation, and that the deletion of Lrrc8d inhibits taurine release [14]. The upregulation of Lrrc8d in TauTKO rats indicated that the upregulation of Lrrc8d may be caused by the deficiency of intracellular taurine.

### KEGG pathway analysis of differentially expressed proteins

In order to further analyze the biological processes involved in differentially expressed proteins, KEGG analysis was performed. We selected the top 24 for analysis (Fig. 3B; Table 3). In terms of biological metabolism, we found that TauTKO and WT rats showed differences in lysine degradation, valine, leucine and isoleucine biosynthesis, pantothenate and CoA biosynthesis and steroid biosynthesis. And lysine degradation pathway may eventually cause the increase of acetyl CoA. The valine, leucine and isoleucine biosynthesis and pantothenate and CoA biosynthesis will promote the synthesis of valine and isoleucine, and the degradation of these amino acids will also increase acetyl CoA. The synthesis of steroid is increased on the basis of the steroid biosynthesis pathway. Acetyl CoA is necessary for steroid synthesis. In conclusion, we concluded that TauT depletion would promote steroid synthesis. In addition, we also found that the lysine degradation pathway decreases the synthesis of carnitine, which plays a role in the transport of fatty acids from cytoplasm into mitochondria during the process of fatty acid. Therefore, the degradation of carnitine inhibits the oxidation of fatty acids. This reinforces the idea above.

**Table 3:** The first 24 KEGG pathways and related proteins involved

Term	P value	Genes
rno00310: Lysine degradation	0.003	Tmlhe, Aass
rno04110: Cell cycle	0.006	Rbx1, Hdac2
rno05220: Chronic myeloid leukemia	0.009	Hdac2, Pik3r2
rno04213: Longevity regulating pathway—multiple species	0.010	Hdac2, Pik3r2
rno0521: Renal cell carcinoma	0.013	Rbx1, Pik3r2
rno00290: Valine, leucine and isoleucine biosynthesis	0.013	Bcat2
rno04066: HIF-1 signaling pathway	0.020	Rbx1, Pik3r2
rno04919: Thyroid hormone signaling pathway	0.020	Hdac2, Pik3r2
rno05206: MicroRNAs in cancer	0.024	Hdac2, Pik3r2
rno00770: Pantothenate and CoA biosynthesis	0.026	Bcat2
rno00100: Steroid biosynthesis	0.026	Sqle
rno04915: Estrogen signaling pathway	0.028	Krt27, Pik3r2
rno05169: Epstein-Barr virus infection	0.030	Hdac2, Pik3r2
rno05200: Pathways in cancer	0.030	Rbx1, Hdac2, Pik3r2
rno05203: Viral carcinogenesis	0.033	Hdac2, Pik3r2
rno04330: Notch signaling pathway	0.044	Hdac2
rno03420: Nucleotide excision repair	0.044	Rbx1
rno04710: Circadian rhythm	0.044	Rbx1
rno05170: Human immunodeficiency virus 1 infection	0.061	Rbx1, Pik3r2
rno05150: Staphylococcus aureus infection	0.065	Krt27
rno04350: TGF-beta signaling pathway	0.065	Rbx1
rno05170: Human papillomavirus infection	0.077	Hdac2, Pik3r2
rno04960: Aldosterone-regulated sodium reabsorption	0.082	Pik3r2
rno05218: Melanoma	0.086	Pik3r2

In the analysis of cell processes in the KEGG pathway, we found that the upregulation of Rbx1 and Hdac2 induced by TauT knockout inhibited the cell cycle. Hdac2 and Pik3r2 can promote biological processes of chronic myeloid leukemia, longevity regulating pathway-multiple species, thyroid hormone signaling pathway, microRNAs in cancer, epstein-barr virus infection and viral carcinogenesis and human papillomavirus infection.The expression of Rbx1 and Pik3r2 promoted the processes of renal cell carcinoma, HIF-1 signaling pathway, and human immunodeficiency virus 1 infection. In addition, the Rbx1, Hdac2, and Pik3r2 are involved in the signaling pathway of cancer cells.

### Validation of differentially expressed proteins.

**Fig. 4:**
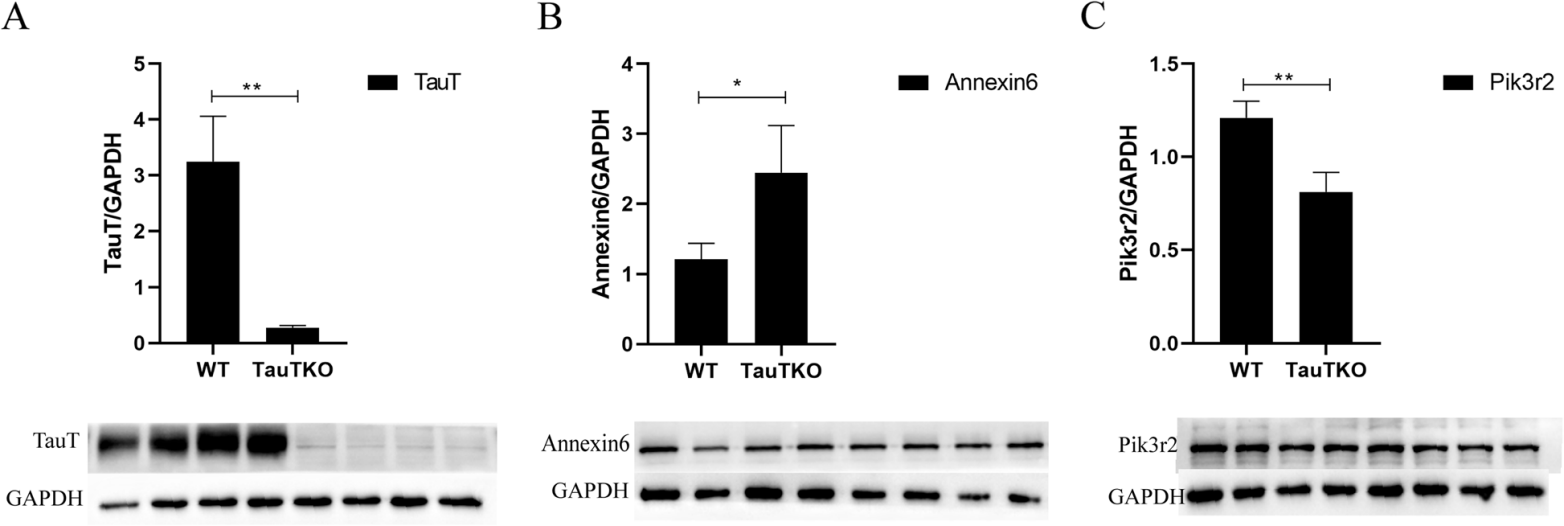
Western blot was used to detect the protein expression. **A** TauT; **B** Annexin6; **C** Pik3r2; *p < 0.05, **p < 0.01 (*n* = 4)

**Fig. 5:**
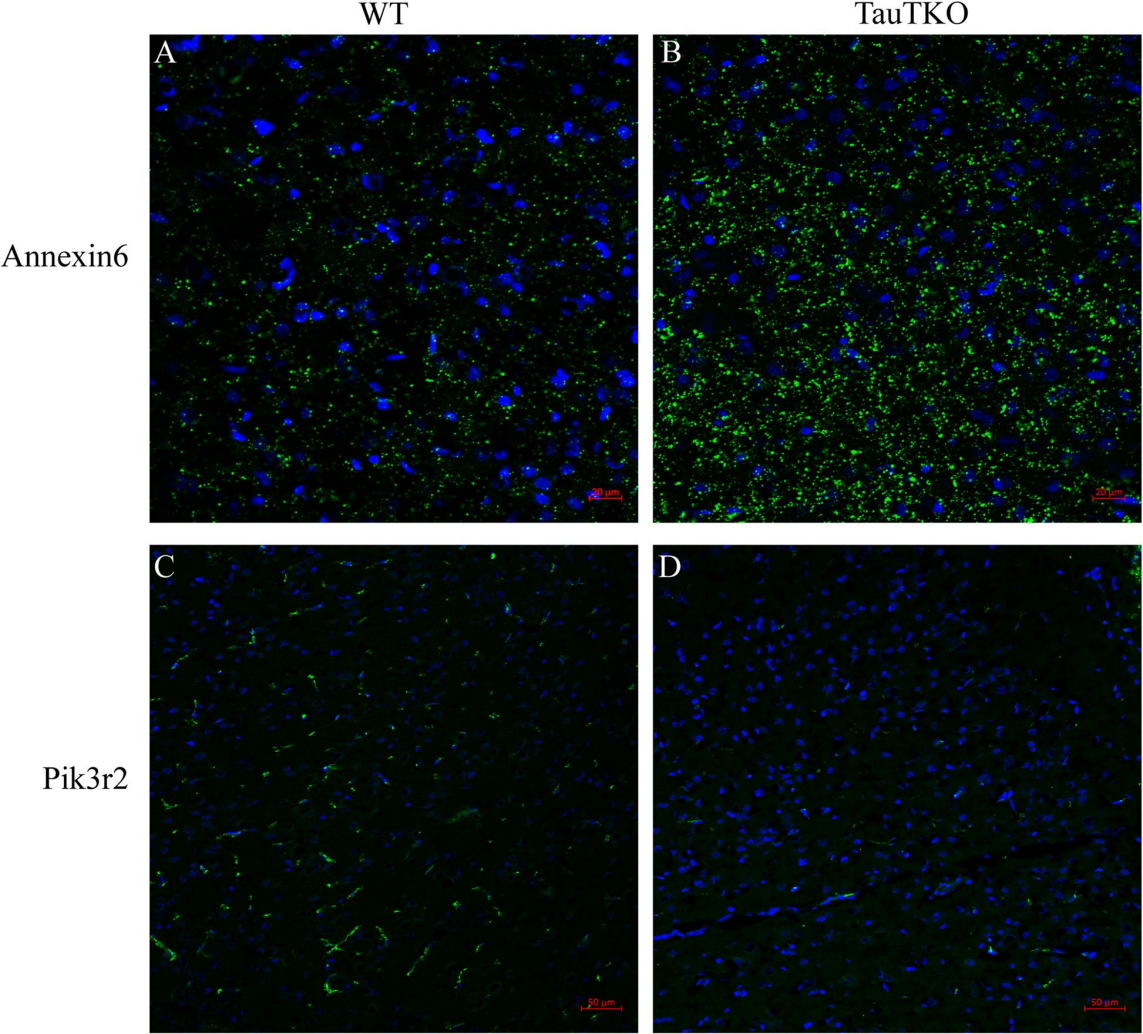
Immunofluorescence was used to detect protein expression. **A**, **B** Annexin6 protein expression in WT and TauTKO rats, respectively; **C**, **D** Pik3r2 protein expression in TauTKO and WT rats, respectively, (200 ×)

Annexin6 and Pik3r2 were selected to confirm the proteomic results. Annexin6 had a high score, while Pik3r2, although not high score, was closely related to the occurrence and development of tumors in the KEGG pathway. TauT knockout induced the upregulation of Annexin6 but inhibited the expression of Pik3r2, which was consistent with the proteomics results (Fig. 4). In addition, the results of immunofluorescence were also consistent with the results of western blotting (Fig. 5).

## Discussion

By screening differentially expressed proteins for study targets, proteomics has been more significant in the application of unknown research. Taurine's role has been intensively researched in the scientific community since 1975 [15]. Finding the mechanism of taurine has grown increasingly challenging, thus the use of proteomics offers a new way to investigate taurine's mechanism.

In this study, We comprehensively analyzed the effect of TauT knockout on cortical protein expression in rats by proteomics, and gained a new understanding of the function of TauT. In the comparative analysis of the proteome of TauTKO rats and WT rats, we found that the TauT knockout did not produce a large number of differentiated proteins, but only 35 differentiated proteins were found. This result may be due to the fact that the TauT itself only acts as a taurine transport channel and is located on the cell membrane, which makes it difficult to participate in intracellular biological processes. Its role is mainly drawn by taurine in the cell.This is demonstrated in the PPI analysis.

Through GO analysis, we understand the involvement of differential proteins in biological processes, cellular components, and molecular functions, but it is difficult to further analyze the correlation of the effects caused by TauT knockout. Therefore, we further analyzed the differential proteins through the KEGG pathway.

Based on KEGG pathway analysis, we first found that the differentially expressed protein involved four metabolic pathways, namely lysine degradation, valine, leucine and isoleucine biosynthesis, pantothenate and CoA biosynthesis and corticosteroid biosynthesis.Lysine degradation involves two proteins, Tmlhe and Aass. N-6-trimethyllysine dioxygenase encoded by Tmlhe is the first step in carnitine synthesis [16].The downregulation of Tmlhe will hinder the synthesis of carnitine. Carnitine plays an important role in the catabolism of long chain fatty acids [17]. And the most common clinical manifestation of carnitine biosynthesis and transport disorders is cardiomyopathy with or without systemic skeletal muscle weakness [18]. This may be related to Ito's report that TauT knockout in mice leads to myocardial and skeletal muscle abnormalities [8]. In addition, the upregulation of Aass protein expression promoted lysine degradation, while the upregulation of Bcat2 protein promoted the catabolism of branched chain amino acids (BCAAs; leucine, isoleucine, and valine) [19]. The catabolism of branched amino acids promotes the lipogenesis [20], this result is the same as our Sqle protein up-regulation promotion on steroids Biosynthesis.

In addition to its metabolic effects, TauT knockout may lead to upregulation of Rbx1 and Hdac2 protein expression. KEGG pathway showed that the up-regulation of Rbx1 and Hdac2 protein expression is related to cell cycle. Han et al. reported that TauT knockout led to cell cycle G2 arrest [21]. This may be due to changes in Rbx1 and Hdac2 protein expression.

In KEGG analysis, we found that Hdac2 and Pik3r2 proteins are involved in many biological pathways, include chronic myeloid leukemia, longevity regulating pathway-multiple species, thyroid hormone signaling pathway, microRNAs in cancer, epstein-barr virus infection, pathways in cancer and viral carcinogenesis and human papillomavirus infection. Overexpression of Hdac2 reduces inflammation, which may indicate that TauT knockout increases inflammatory response [22, 23]. Pik3r2 is an oncogene [24]. In one group of colon and breast cancer samples, Pik3r2 expression levels were elevated in 50% of tumors [25]. TauT is the target gene of the tumor suppressor gene P53, and targeted inhibition of TauT can be used for cancer immunotherapy mediated by P53 mutation [26, 27]. Therefore, inhibition of TauT may inhibit the development of tumor by reducing the expression of Pik3r2.In addition, through proteomic analysis, we found that TauT knockout promoted the expression of Lrrc8d protein. Lrrc8d is a subunit of volume-regulated anion channels (VRACs). The addition of Lrrc8d subunit significantly increased the taurine output and cisplatin input of VRAC [28, 29]. While the platinum-based drugs, such as cisplatin, carboplatin and oxaliplatin are among the most successful in the treatment of cancer [30]. This suggests that inhibition of TauT expression will promote cell uptake of cisplatin. It was consistent with Han's study that TauT overexpression inhibited cisplatin absorption [31].

Annexin6 and Pik3r2 proteins were selectively validated and the results were consistent with proteomics. This demonstrates the reliability of the proteomics results.Annexin6 represents the largest member of the Annexin family. It acts as a multifunctional scaffold protein that recruits signaling proteins, regulates cholesterol and membrane transport, and affects actin dynamics, and it’s up-regulation may have an inhibitory effect on tumor migration [32]. The function of Pik3r2 has been described previously. In addition, our proteomic results showed no down-regulation of TauT protein expression, which is different from our Western blotting results. therefore, we believe that the expression of TauT was not identified as down-regulated, but Slc6a11 in the same family was identified as down-regulated, which may be the limitation of proteomics, and it is not possible to accurately distinguish the fragments with similar peptide sequences in the same family.

In conclusion, our proteomic results not only indicate the importance of TauT for growth and development, but also provide abundant evidence that TauT knockout may affect tumor development from different pathways, which may provide a new field for TauT research.

## Conclusions

In this study, we identified 35 differentially expressed proteins by TauT knockout using proteomics. From these altered proteins, We learned that TauT knockout causes many signaling pathway changes. However, it is worth noting that TauT knockout causes changes in many tumor-related signaling pathways, and these results may help us to explore the effects of TauT on tumors in the future.

## Data Availability

Not applicable.

## Abbreviations

TauT: Taurine transport
HPLC: High performance liquid chromatography
DEPsdiff: Erentially expressed proteins
GO: Gene ontology
PPI: Protein-protein interaction
KEGG: Kyoto Encyclopedia of Genes and Genomes
